# Role of immune cells in the removal of deleterious senescent cells

**DOI:** 10.1186/s12979-020-00187-9

**Published:** 2020-06-03

**Authors:** Abhijit Kale, Amit Sharma, Alexandra Stolzing, Pierre-Yves Desprez, Judith Campisi

**Affiliations:** 1 Buck Institute for Research on Aging, 8001 Redwood Boulevard, Novato, CA 94945 USA; 2 SENS Research Foundation, 110 Pioneer Way, Suite J, Mountain View, CA 94041 USA; 3 Centre for Biological Engineering, Wolfson School of Mechanical, Electrical and Manufacturing Engineering, Loughborough University, Loughborough, UK; 4 California Pacific Medical Center, Research Institute, San Francisco, CA 94107 USA; 5 Biosciences Division, Lawrence Berkeley National Laboratory, Berkeley, CA 94720 USA

**Keywords:** Age-related pathology, Cell-based therapy, Cellular senescence, Immune surveillance, Inflammation, Macrophages, Natural killer cells

## Abstract

Cellular senescence is an essentially irreversible arrest of cell proliferation coupled to a complex senescence-associated secretory phenotype (SASP). The senescence arrest prevents the development of cancer, and the SASP can promote tissue repair. Recent data suggest that the prolonged presence of senescent cells, and especially the SASP, could be deleterious, and their beneficial effects early in life can become maladaptive such that they drive aging phenotypes and pathologies late in life. It is therefore important to develop strategies to eliminate senescent cells. There are currently under development or approved several immune cell-based therapies for cancer, which could be redesigned to target senescent cells. This review focuses on this possible use of immune cells and discusses how current cell-based therapies could be used for senescent cell removal.

## Background

**Fig. 1:**
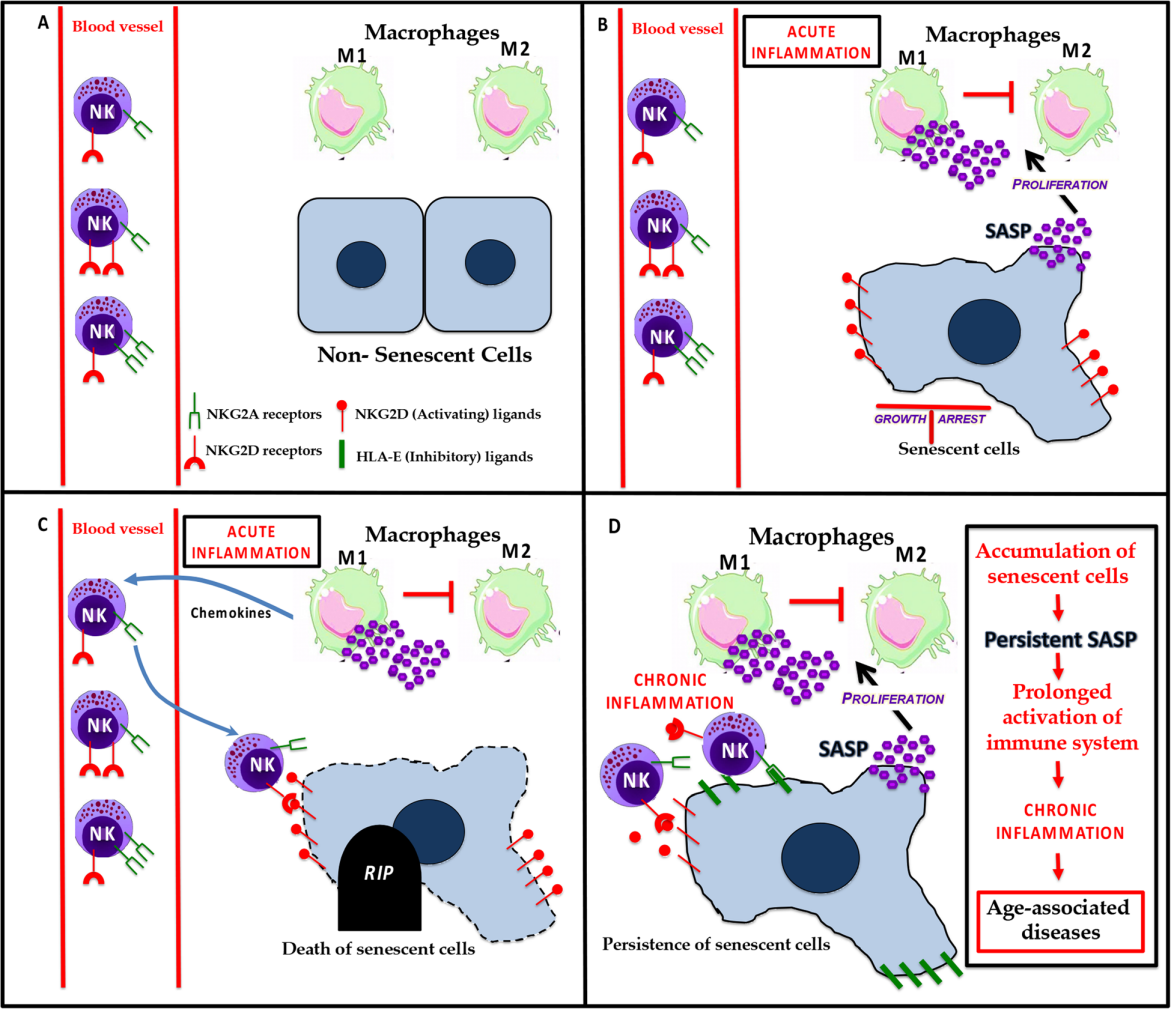
**a** In a normal tissue microenvironment, the diverse populations of cells are healthy. **b** In response to different stressors, some cells undergo irreversible growth arrest and acquire a senescent phenotype. Senescent cells have an effect on the innate immune system by secreting inflammatory factors that are part of the SASP. The SASP generally promotes the proliferation and polarization of M1 macrophages and the suppression of M2 macrophages. **c** In response to the amplified inflammatory signals, NK cells are recruited to site(s) containing senescent cells, which express NK activating ligands on their surface. These NKG2D ligands bind to NKG2D receptors present on NK cells leading to the death of senescent cells. **d** However, some senescent cells have strategies to avoid elimination. For example, they can express inhibitory ligands that bind to NKG2A receptors on NK cells, blocking their killing. The immune evasion of senescent cells can lead to their accumulation in tissues over time and causes age-associated diseases

Cellular senescence entails an essentially irreversible arrest of proliferation in damaged or stressed cells that are at risk of malignant transformation [1, 2]. Two main pathways establish and maintain this growth arrest, which is a potent anti-cancer mechanism. One pathway is governed by p53 (a tumor suppressor and transcriptional regulator) and p21 (a cyclin-dependent kinase (CDK) and cell cycle inhibitor). The other pathway is governed by p16^INK4a^ (a tumor suppressor and CDK/cell cycle inhibitor) and pRB (a tumor suppressor and transcriptional regulator). Several stimuli can trigger these pathways, leading to senescence in cultured cells and in vivo [3]. Important stimuli for senescence include replicative exhaustion, which generally results in telomere attrition (also known as replicative senescence) [4], and DNA-damage such as that caused by ionizing and, to some extent, non-ionizing radiation [5]. In addition, some chemotherapeutic drugs such as doxorubicin or bleomycin also cause DNA damage, and other drugs such as abemaciclib or palbociclib inhibit CDKs directly to induce a senescence arrest [6]. Consistent with senescence being an antitumor mechanism, the activation of certain oncogenes such as RAS or BRAF leads to oncogene-induced senescence (OIS) [3, 7]. Further, events that disrupt mitochondrial function triggers a mitochondrial dysfunction-associated senescence (MiDAS) arrest [8], and oxidative stress [9, 10] and inhibitors of DNA methylases or histone deacetylases [6] also cause a senescence arrest (Fig. 1).

The senescence arrest is generally coupled to a complex senescence-associated secretory phenotype (SASP) [11]. The SASP is conserved between mice and humans [12], and even to some extent between mammals and insects [13]*.* Its components include growth factors, chemokines and cytokines, proteases, bioactive lipids and extracellular vesicles, many of which are pro-inflammatory [14]. The number of senescent cells increases with age in most tissues, although they rarely exceed a few percent. Nonetheless, mounting evidence suggests that senescent cells can drive a surprisingly diverse array of aging phenotypes and diseases, mainly through the SASP [8, 15–19]. The presence of senescent cells also exacerbates several diseases including, but not limited to, osteoarthritis [20], osteoporosis [21], atherosclerosis [22], Parkinson’s disease [23], and Alzheimer’s disease [24, 25]. Importantly, eliminating senescent cells in transgenic mouse models often delays age-related tissue dysfunction and increases health span [26]. Furthermore, several laboratories are developing new classes of drugs termed senolytics, which kill senescent cells, or senomorphics, which alleviate SASP effects. These drugs can help maintain homeostasis in aged or damaged tissues, and postpone or ameliorate many age-related pathologies [21, 23, 24, 26–30].

In contrast to their deleterious roles in driving aging and age-associated diseases, senescent cells can have beneficial roles during development and tissue repair, regeneration and reprogramming. For example, in mice, the SASP from senescent cells enhances reprogramming in neighboring cells, and the short-term expression of reprogramming factors promotes tissue regeneration and reduces tissue aging [31, 32]. Senescent cells can also promote wound healing in the skin and liver, and either promote or suppress fibrotic responses depending on the tissue and biological context [29, 33–37]. Senescent cells also optimize mouse embryogenesis, and the absence of senescent cells can delay development and promote patterning defects [38, 39]. In adult animals, senescent cells promote heart regeneration, and their elimination can impair regeneration and repair in this tissue [40, 41].

Current thinking is that the short-term presence of senescent cells is beneficial, largely by adjusting the plasticity of neighboring cells, but that their prolonged presence can be deleterious. This apparent dichotomy of the impact of cellular senescence on health and disease suggests that cellular senescence is an example of antagonistic pleiotropy, the evolutionary theory that predicts there are traits that have been selected for their beneficial effects early in life, but late in life these traits can be maladaptive and drive phenotypes and pathologies associated with aging [42].

The timely clearance of senescent cells is required to maintain tissue and organismal homeostasis. Although cellular senescence has been studied in detail in the context of disease, the interaction of senescent cells with immune cells have been less thoroughly investigated. Due in large measure to the SASP [11, 14], senescent cells likely interact extensively with the immune system [43]. The production and secretion of SASP factors (resulting in local inflammation) can be a potent means to recruit immune cells. The SASP recruits macrophages, natural killer (NK) cells, neutrophils and T lymphocytes, which eliminate them, but senescent cells can also interact with immune cells to avoid elimination.

The immune system was first shown to eliminate senescent cells in a study demonstrating that reactivation of p53 in hepatic tumors causes the tumor cells to senesce, followed by ‘selective’ recruitment of macrophages, neutrophils and NK cells by the SASP-producing senescent cells [44]. Subsequently, p53 was shown to promote the secretion of chemokines like CCL2 to attract NK cells for the clearance of senescent cancer cells [45]. A role for the SASP in immune clearance of senescent cells was further highlighted by the finding that the epigenetic regulator BRD4, which dictates the enhancer and super-enhancer landscape of SASP genes, determines the ability of the SASP to promote immune clearance of senescent cells [46]. Thus, BRD4 inhibition significantly reduces the SASP, which severely limits the ability of the immune system to eliminate senescent cells. Further, expression of the scavenger receptor CD36 is sufficient to induce a SASP in normal dividing cells, suggesting an important role for this receptor in SASP signaling [47]. Here, we first describe the function of various cell types of the immune system, and then discuss possible therapies for the elimination of senescent cells by immune cells.

### Interaction of senescent cells with macrophages

Monocytes-macrophages belong to a class of multifunctional innate immune cells prevalent throughout the body, and maintain tissue homeostasis and repair by regulating various biological processes such as angiogenesis and tissue remodeling [48–50]. These innate immune cells recognize and eliminate bacterial pathogens based on pathogen-specific molecular patterns [51]. Thus, macrophages are important players in resolving infections. They also can promote certain diseases such as asthma, rheumatoid arthritis, cancer and atherosclerosis [52].

These antigen-presenting cells are classically divided into two phenotypically distinct subgroups (M1 and M2), but with a high degree of plasticity somewhat similar to the Th1 and Th2 classification assigned to T cells [49, 50]. Exposure to intracellular pathogens and their components, such as lipopolysaccharide (LPS) or cytokines such as interferon (IFN)-γ, can trigger an M1-type phenotype in macrophages, which produce ‘pro-inflammatory’ molecules. These molecules include interleukins (IL)-1β, IL-6, IL-8, IL-12 and IL-15, tumor necrosis factor (TNF)-α and chemokines to enhance the clearance of pathogens [49]. M1-like cells also show increased major histocompatibility complex (MHC) class II expression [53], and increased inducible nitric oxide synthase activity [48].

M2-type macrophages are phenotypically distinct macrophages that respond to type II cytokines such as IL-4 and IL-13, in addition to counteracting the effects of IL-1β signaling [54]. These cytokines upregulate arginase-1, which shifts metabolism to reduce nitric oxide production but increase polyamine and proline production, which stimulate cell growth, dynamic changes in collagen and tissue repair [55]. M2-type macrophages also produce high levels of IL-10 and matrix metalloproteinase (MMP)-12, as well as chemo-attractants such as CCL-8 and CCL-13 to attract eosinophils and basophils to resolve tissue damage.

One primary function of macrophages is resolution of inflammation by ‘clearing’ culprit damaged cells. The role of macrophages in eliminating senescent cells was first demonstrated a decade ago [56]. In mice, macrophages clear senescent cells in the uterine stroma surrounding the embryo implantation site following parturition [57]. In addition, senescent hepatic stellate cells secrete a SASP that not only attracts macrophages [58], but also converts cytotoxic M1 cells to the M2 state [59]. Stellate cells undergoing senescence preferentially secrete IL-6, ICAM1 and IFN-γ, triggering M1 polarization, whereas proliferating stellate cells secrete IL-3, IL-4, and IL-5, among other factors, which shift macrophages to the M2 state [59]. Aging diminished the ability of macrophages to respond to a cytokine that shifted resident macrophages to an M1 state but caused paradoxical IL-4-driven polarization of resident macrophages toward the M1 state [60]. The expression of p16^INK4a^ in macrophages can suppress M1 polarization and hence the secretion of inflammatory factors by these cells [61]. On the other hand, the SASP secreted by senescent thyroid cells skews macrophage polarization to M2 caused by prostaglandin E2 [62], a prominent SASP factor [63].

There are several unanswered questions regarding the interplay between senescent cells and macrophages and how this interplay influences age-related inflammation or what is now termed inflammaging [64]. Recent findings in murine models show that some cells with elevated p16^INK4a^ and senescence-associated beta-galactosidase (SA-β-Gal) expression (common biomarkers of senescence) are likely macrophages, and that these macrophages exhibit other phenotypes associated with cellular senescence [65]. These senescent-like macrophages increase with age, and might exacerbate the rise in senescent cells and the SASP during aging [66] by a paracrine effect, which was recently shown to occur in vivo [67, 68]. Furthermore, eliminating this subset of so-called senescent-like or pseudo senescent macrophages with an M2 phenotype induces a striking resolution of inflammation [69]. Recent work also shows that the SASP can promote macrophage proliferation and increased expression of CD38, which enhances the consumption of NAD by macrophages and might explain the age-related decline in NAD levels [70].

M1 and M2 macrophages are still difficult to distinguish [71]. There is a consensus that macrophages can switch their phenotypes to those associated with M1 and M2 states in response to different microenvironments [72]. Indeed, M1 and M2 macrophages vary in phagocytic activity in response to their microenvironment [73, 74]. Thus, macrophages and senescent cells may interact depending on the specific SASP signature and ligands present on senescent cells, which in turn depends on the lineage of cells undergoing senescence and the nature of the insult responsible for inducing senescence [14] (Fig. 1).

### Interaction of natural killer cells with senescent cells

NK cells were originally described as ‘Large Granular Lymphocytes’ with natural innate ability to kill cancer cells [75]. Since then, these innate immune cells were shown to eliminate aberrant cells, including virally infected cells, ‘stressed’ cells and cancer cells without prior stimulation or activation [75]. NK cells appear to function primarily by surveilling MHC class I expression. This function prevents the activation of NK cells against ‘self’ cells, but the lowering of MHC class I on damaged or cancer cells allows NK cells to eliminate such cells as a first line defense against aberrant cell proliferation and cancer [76]. In humans, MHC class I molecules are recognized by a family of receptors called killer cell immunoglobin-like receptors (KIR) [77], which can either activate or inhibit NK cell killing [78]. Another important receptor, natural killer group 2A (NKG2A) (CD94), binds to the ubiquitously expressed HLA class I molecule HLA-E to suppress NK cell cytotoxicity [79]. On the other hand, several activating receptors, such as natural killer group 2D (NKGD2) [80], or DNAX accessory molecule-1 (DNAM-1) [81], increase on NK cells upon interaction with stressed cells. NK cells are now characterized based on the expression of the specific receptors that fine-tune NK cell-mediated cytotoxicity [82].

The differential expression of CD56 has most often been used to identify NK cells in humans. Low (CD56^dim^) and high (CD56^bright^) CD56 expression levels define major subsets, along with an absence of CD3. CD56^bright^ CD16^−^ cells are considered immature NK cells that secrete IFNγ, whereas CD56^dim^ CD16^+^ NK cells are responsible for cytotoxicity [83]. Upon physical interaction with target cells, cytotoxic NK cells release perforin, granzymes (serine proteases) and proteoglycans contained in cytotoxic granules that kill the target cells [84].

One important consequence of the SASP is the attraction of NK cells [45]. NK cell-mediated clearance of senescent cells is an essential aspect of tissue homeostasis [85, 86] and tumor growth limitation [44, 87]. Impairing NK cell function results in an accelerated accumulation of senescent cells in various tissues, at least in animal models [88]. Furthermore, aging can alter NK cell cytotoxicity and cytokine production [89]. Although NK cells from different individuals vary substantially in their expression of surface receptors [90], the proportion of CD56 ^dim^ NK cells increases with age [91], and cytokine-producing CD56^bright^ NK cells decline after age 60 years [92].

DNA damage is known to induce the expression of several NK cell receptors, such as NKG2D and DNAX accessory molecule-1 (DNAM-1) ligand, on target cells [93]. This induction has been utilized to increase the immune clearance of multiple myeloma, where induction of senescence upon treatment with genotoxic chemotherapeutic drugs like Doxorubicin enhances NK cell-mediated elimination of cancer cells due to increased expression of DNAM-1 [94–96]. In addition, recent studies show that senescent cells acquire mechanisms to evade clearance by NK cells. For instance, senescent fibroblasts in culture and in the skin of older humans increase expression of HLA-E, which interacts with NKG2A to inhibit NK cytotoxicity [97]. Senescent cells can also shed MICA and MICB, which are ligands for NKG2D receptors expressed on NK cells and are primarily responsible for NK cell targeting of senescent cells [98]. The shedding of these ligands by metalloprotease secretion as part of the SASP [99] can prevent NK cells from binding to target cells [98] (Fig. 1).

### Interaction of other immune cell types with senescent cells

T cells are a type of lymphocyte that play a central role in adaptive immune responses. These thymus-derived cells mature by interacting with foreign antigens presented, along with MHC molecules, on antigen-presenting cells (APCs) through their T cell receptors. Depending on the microenvironment, these cells can mature into cytotoxic CD8^+^ cells that aid in the maturation of B cells upon subsequent interaction with pathogenic antigens presented by APCs, helper CD4^+^ memory cells, or natural killer T (NKT) cells [100]. CD8^+^ T cells can also target senescent cells by interacting with NKG2D ligands (described above) [97]. CD4^+^ T cells are required for proper macrophage-dependent elimination of senescent hepatocytes (induced by oncogene expression) in vivo, suggesting that Th1 lymphocytes participate in immune surveillance of senescent cells [101]. Further, oncogene-induced senescence in melanocytes can activate CD4^+^ T cell proliferation, concurrent with increased MHC II expression on senescent cells, suggesting recruitment of the adaptive immune system to prevent tumor growth [102]. Finally, neutrophils mediate the immediate host response to bacterial and fungal infections. Along with NK cells and macrophages, neutrophils also infiltrate tissues containing senescent cells [44], and are susceptible to age-dependent decline in numbers and phagocytic function [103].

### Potential therapies for the immune clearance of senescent cells

There are currently several immune cell therapies for cancer under development or approved, which could potentially be redesigned to target senescent cells. Cell-based therapies have greatly improved in recent years with the optimization of cell production, cell modifications, and storage [104]. The following section focuses on how current cell therapies could be employed for senescent cell removal.

#### Therapeutic use of CAR-T cells

Chimeric antigen receptor (CAR) T cell therapy has been successful in recent years for treating diseases such as cancer. CAR-T cell therapy uses autologous cells that are genetically modified ex vivo to encode a synthetic receptor that binds a known antigen [105]. The modified cells are then infused back into the patient to kill the target cells. A universal CAR-T cell product could eliminate many of the harvesting and manufacturing problems associated with autologous or HLA matched CAR-T cells. Advantages of CAR-T cells over other cell types include their capacity to induce durable responses and their ability to override tolerance to self-antigens [106]. CAR-T cell targeting moieties are not restricted to antibody targets, as non-antibody structures such as aptamers and polypeptides have been used [107]. However, a potential downside to this approach is that some antigens used to target cancer cells are also present in healthy tissues, albeit generally at much lower levels [108].

Evidence that there are senescent-specific surface markers is spotty [43], and specificity needs further validation. Nonetheless, once a good target has been identified, it can be used to create a CAR-T cell. Alternative CAR-T strategies are being developed to improve specificity or effectiveness that could be helpful in the context of senescent cells. One such alteration is the use of several antigens for improved recognition [107], allowing more specific recognition of senescent cells. The therapeutic potential of CAR-T cells in targeting senescent cells stems from their success in the treatment of solid tumors, as CAR-T cells are observed to reach deep into the parenchyma of many different organs in which senescent cells reside.

#### Therapeutic use of natural killer cells

As senescent cells are naturally targeted for elimination by NK cells, it could be beneficial to use NK cells to eliminate persistent pro-inflammatory senescent cells, particularly as they accumulate during aging. The broad cytotoxicity and rapid killing ability make NK cells ideal for use in cancer immunotherapy. Indeed, long before the era of CAR-T cells, researchers used NK cells to fight cancers [109]. NK cells have been an attractive choice for allogeneic immunotherapy for various cancers such as acute myeloid leukemia, and can be easily isolated and enriched from a variety of sources like peripheral blood, bone marrow or cord blood. NK cells isolated from healthy young donors are not only fully functional, but can also eliminate cancer cells by robust graft-versus tumor response as they do not express inhibitory receptors specific to host cells [110]. Even though, technical, logistical and financial challenges are still limiting factors for applications of circulating NK cells as promising cancer therapies, over the past decade, several studies demonstrated the safety and efficacy of allogeneic NK cells against various hematological malignancies and, to a lesser extent, solid tumors [111]. Further, induced pluripotent stem cells have been genetically modified with an NK-CAR construct and differentiated into NK cells. These cells were tested in a mouse tumor model and were effective at eliciting a lower cytokine level in recipients, indicating that these cells might be safer [112], given that repeated administration is needed. The same technology could be used to target senescent cells by NK cells.

An additional benefit of NK-CAR cells over CAR-T cells is that the former retains their ability to recognize target cells through their native receptors, making it less likely for tumor cells to escape by downregulating the CAR target antigen. NK-CAR cells do not undergo clonal expansion or quick immune rejection [113]. NK cells do not require strict HLA matching and lack the potential to cause graft-versus-host disease, an important risk imposed by CAR-T cell therapy. NK-CAR cells could therefore be an off-the-shelf allogeneic therapeutic for the effective elimination of pro-inflammatory senescent cells. On the other hand, senescent cells can escape NK-mediated killing by overexpressing MMP3 (which cleaves activating MICA ligands from the senescent cell surface) [98] or HLA-E (an inhibitory ligand that blocks NK cell killing) [97]. Unfortunately, MMP-3 inhibitors have serious side effects and thus are not generally useful.

#### Therapeutic use of macrophages

As discussed above, macrophages can eliminate senescent cells. Transplanted macrophages can migrate into tissues and become tissue-resident with much longer half-lives and self-renewal abilities [114]. Targets for macrophage cell therapies are more numerous than other cell types and potentially include cancers, myocardial infarcts, osteoporosis and Alzheimer’s disease [115]. Indeed, because macrophages are phenotypically plastic, and cancer cells often express a “don’t eat me” signal, these therapies have not been very successful in treating cancer [115]. Whether this limitation poses a difficulty in using macrophages against senescent cells is not clear. Further, NFκB-dependent pro-inflammatory signaling appears to upregulate CD47, at least in some cancers, facilitating their escape from immune surveillance [116]. Senescent cells generally upregulate NFκB activity, which can activate CD47 transcription [117]. As a cell surface molecule that promotes immune evasion by engaging signal-regulatory protein alpha (SIRPα), CD47 serves as an inhibitory receptor on macrophages [118]. It is possible that some senescent evade macrophage-mediated killing by increasing CD47 signaling, in some cases by secreting its ligand, thrombospondin. Notably, some macrophages have been engineered to lack the SIRPα co-receptor [119] to overcome this evasion. Moreover, allogeneic macrophages from young donors or induced pluripotent stem cells (iPS) [120] would probably be more effective at removing senescent cells, as they have a higher phagocytosis capacity [121].

## Conclusions

A better understanding of the interplay between immune cells and senescent cells would illuminate changes that happen during aging, and also speed the development of novel therapeutic interventions for eliminating deleterious senescent cells. Different approaches could be formulated to remove senescent cells using the natural ability of immune cells. What is needed now is a more thorough understanding of the heterogeneity of senescent cells and of the specific targets for immune cells. In addition, it will be important to determine how tissue resident macrophages interact with senescent cells, and whether the propagation of paracrine senescence increases the senescent cell burden. Finally, it will be critical to understand the mechanisms by which senescent cells escape immune clearance.

## Data Availability

Not applicable.

## Abbreviations

SASP: Senescence-associated secretory phenotype
CDK: Cyclin-dependent kinase
OIS: Oncogene-induced senescence
MiDAS: Mitochondrial dysfunction-associated senescence
NK: Natural killer
LPS: Lipopolysaccharide
IFN: Interferon
IL: Interleukin
TNF: Tumor necrosis factor
MHC: Major histocompatibility complex
MMP: Matrix metalloproteinase
SA-β-Gal: Senescence-associated beta-galactosidase
NKG2A: Natural killer group 2A
APCs: Antigen-presenting cells
NKT: Natural killer T cells
CAR: Chimeric antigen receptor
SIRPα: Signal-regulatory protein alpha
iPS: Induced pluripotent stem cells
